# Ontological Constraints in Children's Inductive Inferences: Evidence From a Comparison of Inferences Within Animals and Vehicles

**DOI:** 10.3389/fpsyg.2018.00520

**Published:** 2018-04-30

**Authors:** Andrzej Tarlowski

**Affiliations:** Department of Cognitive Psychology, University of Finance and Management in Warsaw, Warsaw, Poland

**Keywords:** concepts, categories, inferential reasoning, naive biology, domain knowledge, perceptual similarity

## Abstract

There is a lively debate concerning the role of conceptual and perceptual information in young children's inductive inferences. While most studies focus on the role of basic level categories in induction the present research contributes to the debate by asking whether children's inductions are guided by ontological constraints. Two studies use a novel inductive paradigm to test whether young children have an expectation that all animals share internal commonalities that do not extend to perceptually similar inanimates. The results show that children make category-consistent responses when asked to project an internal feature from an animal to either a dissimilar animal or a similar toy replica. However, the children do not have a universal preference for category-consistent responses in an analogous task involving vehicles and vehicle toy replicas. The results also show the role of context and individual factors in inferences. Children's early reliance on ontological commitments in induction cannot be explained by perceptual similarity or by children's sensitivity to the authenticity of objects.

## Introduction

A lively debate on the early development of induction is motivated by two opposing views, namely the *perceptual* and the *early knowledge* view. While both approaches agree that perceptual information informs children's induction (Gelman and Medin, 1993; Jones and Smith, 1993), the controversy lies in the role of category knowledge in children's inferences. By the perceptual view, until about 6 years of age, induction is driven purely by perception and does not employ category knowledge. The reliance on categories is a late achievement requiring maturation, accumulation of knowledge, explicit training, and cognitive resources (Sloutsky and Fisher, 2004; Fisher and Sloutsky, 2005; Sloutsky et al., 2007; Sloutsky, 2010; Badger and Shapiro, 2015). According to the early knowledge view, young children's inferences employ rich category knowledge which is embedded in domain theories (Murphy and Medin, 1985). When making inferences, children factor in broad, abstract, unobservable properties of categories which derive from their rudimentary understanding of the domains to which the categories belong. Even very young children construct intuitive ontologies, that is, partition their experience into basic categories of existence (Keil, 1979; Carey, 1985). Ontologically basic categories are picked out by large clusters of predicates and terms (Carey, 1985). For example, “is alive, breathes, has babies, can die, is ill” all apply to animals. Ontological categories are diagnosed through a linguistic test. If a predicate is inappropriately applied across the ontological boundary, it generates category mistakes, e.g., “a car has babies, a rock is ill,” statements that are nonsensical rather than simply false (Carey, 1985). Children's ontological commitments, for example, an intuition that animals, but not artifacts have essences, contribute to differences in inference patterns within natural kinds and artifacts that cannot be explained by perceptual properties of the stimuli (Gelman and Markman, 1986; Keil, 1989; Keil et al., 1998; Gelman, 2003; Gelman and Davidson, 2013).

The conflicting construals of the role of categories in induction are related to accounts of the origins of knowledge. The perceptual view is associated with the empiricist tradition claiming that statistical learning from experience fully explains the development of knowledge systems (Sloutsky, 2010). The early knowledge view stems from a variety of accounts, which typically postulate either domain specific (Medin and Atran, 2004; Carey, 2009) or domain general (Keil, 1994; Gelman, 2003) innate constraints on knowledge acquisition.

### The evaluation of the theoretical views on the role of categories in induction

The primary challenge for the research evaluating perceptual and conceptual influences on induction involves striking the right balance between tight control of perceptual similarity and the quality of conceptual information. Category membership is correlated with perceptual similarity and disentangling the two poses methodological problems. Gelman and Markman's (1986) classic solution was to draw from real life cases, in which perceptual information conflicts with conceptual information, for example, children made inferences from either a flamingo or a bat to a swallow. Gelman and Markman (1986) showed that, when making inferences, children ignored perceptual similarity and relied on category membership. However, Sloutsky and Fisher (2004), argued that labels presented by Gelman and Markman (1986) to signal category membership should be construed as salient perceptual features, rather than cues to category membership. Gelman and Markman's (1986) category-consistent choices can be viewed as perceptual choices if labels are treated as features contributing to perceptual similarity metrics. Sloutsky et al. (2007) provided additional evidence questioning the role of conceptual information in early induction. Instead of relying on real objects they ensured tight control over perceptual similarity by designing novel categories of hypothetical animals. They presented children with two categories of bugs, which were perceptually distinguished by the ratio of buttons to fingers. 4–5-year-olds readily learned to categorize these bugs. However, when making inferences, they ignored category membership, even if it was not pitted against perceptual similarity. Badger and Shapiro (2012) reported a similar result. They also employed hypothetical categories of bugs and additionally portrayed them as more natural than those presented by Sloutsky et al. (2007).

In defense of early knowledge view, it can be argued that Sloutsky et al. (2007) or Badger and Shapiro (2012) did not provide children with conceptual information of sufficient “quality” to support induction (Gelman and Waxman, 2007; Gelman and Davidson, 2013). Conceptual information only supports induction if it resonates with the child's existing knowledge. Gelman and Davidson (2013) and Booth (2014) manipulated the quality of conceptual information and demonstrated children's reliance on categories only when category distinctions marked conceptually coherent and informative groupings. In response to these findings, Sloutsky et al. (2015) argued that Gelman and Davidson's (2013) design confuses attentional factors with conceptual factors and only the attentional factors guide inductions in their design.

Fisher et al. (2015) proposed the Perceptual and Representational Similarity (PaRS) model to reconcile conflicting data on the role of conceptual knowledge in induction. PaRS posits that, unlike mature inferences, children's inferences rely exclusively on similarity metrics which tallies perceptual features and all the semantic knowledge about the entities, acquired through direct or indirect experience. They propose to distinguish between two terms referring to inferences. Describing inferences as category-consistent would make reference to the outcome of inference (projections within a category) without committing to the underlying process. Describing inferences as category-based would imply an assumption that category-consistent inference was based on processing categories.

PaRS emphasizes the role of experience and is supported by data linking individual differences in the semantic organization to the pattern of inferences. Unlike the perceptual view, PaRS model includes conceptual knowledge in the inference-making process, but unlike early knowledge view, it claims that an ability to factor in abstract category information is a late developmental achievement.

From this review of the debate, it seems that early development of inductive inference is too complex to be exhausted by dichotomous arguments on whether children can or cannot rely on conceptual information. Sutherland and Cimpian (2017) deem attempts to exclude category knowledge from accounts of inductive inference as counterproductive. Instead, they suggest, efforts should be made to recognize specific conditions which facilitate or hinder the reliance on categories in induction.

Several factors contribute to the reliance on category knowledge in induction. These include, among others, the scope of the category (e.g., whether it is a basic level or a superordinate category), the type of information that signals the category (the use of category labels, highlighting category specific features, such as eyes, or movement), the ontological domain (e.g., whether the category is an animal or an artifact), the quality of perceptual information (whether the items are presented as real objects, videos, photos, drawings), the prior knowledge of the category (whether the items are familiar, novel or contrived). In what follows, I will discuss research that relates to some of the abovementioned factors, paying particular attention to questions that are still unanswered.

### Category scope

It is well established that inductive power is highest at the basic level and decreases with increasing category scope (Coley et al., 1997). Most studies probe children's inferences within basic level categories (Gelman and Markman, 1986; Sloutsky et al., 2007; Badger and Shapiro, 2012). Only a few probe superordinate level inferences. In open-ended induction tasks, participants are allowed to project the feature to any one of the target items from a large array. Results from these tasks suggest that children's inferences are mostly constrained to basic level categories (Carey, 1985; Gelman and O'Reilly, 1988). Triad induction tasks are more suited to test relatively weak projections within superordinates. Gelman and Davidson (2013) used Sloutsky et al. (2007) triad induction stimuli to test inferences across ontological boundaries. They show that children project from an animate base to another animate target, rather than to a more similar artifact. However, Sloutsky et al. (2015) point out that Gelman and Davidson (2013) specifically draw children's attention to features signaling the ontological distinction, and therefore the category-consistent inference is a result of attentional rather than conceptual factors. It, therefore, remains to be established whether children would make category-consistent projections spontaneously for stimuli, whose animacy or lack thereof were not explicitly highlighted.

Superordinates mark ontological boundaries, they divide entities based on a set of central features, such as being alive or being made by a human. Superordinate-level projections have significant influence on the formation of knowledge, for example, attributing a feature to all animals has a much higher influence on the state of knowledge than attributing it to robins. It is, therefore, crucial to obtain more data on whether children can rely on superordinates in induction, particularly when they reason with realistic objects, and when their attention is not specifically drawn to the item's membership in the superordinate category.

### Domain differences

The early knowledge view claims that children's inductions are driven by high-level abstract expectations about broad classes of objects (Keil, 1989; Keil et al., 1998; Gelman, 2003). Children expect animates to share internal commonalities, while they expect artifacts to share function and to be internally varied (Keil et al., 1998). Diesendruck and Peretz (2013) showed that when perceptual similarity conflicted with information about internal commonality, 5-year-olds tended to rely on internal commonality more when making categorizations of animals than of artifacts. At the same time, when perceptual similarity conflicted with intentional information (category membership intended by the creator), children relied on intentional information for artifacts and not for animals. The question remains, though, whether these domain differences are reflected in children's inferences. Direct comparisons of inductive inferences between domains are not conclusive. Gelman and O'Reilly (1988) showed no differences between living kinds and artifacts in proportion of inductions within the basic level and superordinate categories. They did show a more subtle effect, namely, compared to artifacts, animates received more category-consistent patterns of responses (that is, “those in which the superordinate category could be the basis of the inferences” Gelman and O'Reilly, 1988; p. 881).

Badger and Shapiro (2015) also compared inferences within animals and artifacts. They used artificial stimuli to ensure tight control over perceptual similarity across domains. They showed that the tendency to make category-consistent inferences does not vary by domain, but it is affected by the category structure. Children shift from similarity-based^1^ to category-consistent inductions earlier, if category structure is featural (category difference marked by a single feature value) rather than relational (category distinction marked by a relation between a number of feature values). Based on these findings, Badger and Shapiro argue that the development of inductive inference proceeds in a domain-general fashion, and young children are not able to employ category knowledge in induction. Badger and Shapiro's (2015) findings are compelling, but they have some limitations. Unlike (Gelman and O'Reilly, 1988), who used multiple instances of natural kinds and artifacts as stimuli, Badger and Shapiro relied on a single basic-level distinction within each domain (rocky bug vs. desert bug, town trudge vs. country trudge). It could be the case that their results do not generalize to other basic-level distinctions, for example, it is possible that vertebrate species elicit more category-consistent responses than insect species. Moreover, Badger and Shapiro's (2015) stimuli are artificial and it is not certain that the findings would be replicated with real-life entities (Gelman and Waxman, 2007).

### Inferences to real objects and representations

While developmental theories of inductive inference describe how children reason about real entities, empirical studies present these entities as representations, such as videos (Jipson and Gelman, 2007), realistic photos (Tarlowski, 2006) or drawings of existing objects (Gelman and Markman, 1986), as well as depictions of inexistent entities (Sloutsky et al., 2007; Badger and Shapiro, 2012, 2015). Relying on representations is an obvious and well justified methodological choice. Children's experience is filled with representations (movies, books, toys). Toddlers understand the representational status of photographs (Ganea et al., 2009). Preschoolers extend knowledge gained from picture books to real entities (Ganea et al., 2011). Using representations allows researchers to control the extent of overlap between reality and the stimuli. Representations that are far removed from reality (e.g., depictions of inexistent entities) enable researchers to perform clean tests of category-consistent induction by entirely decoupling category membership from perceptual similarity. At the same time, generalizations from children's performance with such representations are always susceptible to criticism for low ecological validity (Gelman and Waxman, 2007).

There is a way to use representations to experimentally decouple category membership from perceptual similarity without making a substantial departure from reality. It involves testing inferences to toys. Toys are designed to perceptually resemble their referents but they do not belong to the same category (e.g., a bear is an animal, a teddy bear is a toy). Toys are viable experimental targets because, unlike images, they are 3D objects that support projections of physical properties or behaviors. Inferences to toys could provide a strong test of the early knowledge view, which argues that children's inferences observe ontological constraints. For example, Keil (1989) showed that 5-year-olds do not believe it is possible to transform a toy bird into a real bird. Based on this expectation children should resist inferences from animates to toys even if their similarity is compelling, because toy animals, being artifacts, belong to a different ontological category than real animals. So far, there are few studies that test this prediction, and their findings are contradictory. Tarlowski (2006) looked at how parental expertise in biology relates to children's inductive inferences in a task involving projections of internal features from animates to an array of objects including toy representations, all presented on photos. While children of experts differentiated between same basic-level target and same basic-level toy, laypeople's children did not, that is, they were as likely to project from a horse to another horse as to a rocking horse. The negative result observed for children with the standard profile of experience with biological kinds could reflect these children's difficulty reasoning about photographs of toys (which are representations of representations), although Massey and Gelman (1988) showed that even 3-year-olds consider statues presented on photos as inanimate objects.

Carey (1985) showed that children made few projections of known biological features from an animal to a toy monkey (which was presented as a real object, while the other stimuli were images) only when the task involved unfamiliar target animals. Children made projections to the toy monkey just as often as they projected to familiar animals.

Even those responses that reject toys as possessing animate features cannot be readily attributed to children's ontological commitments because Bunce and Harris' (2013) data suggest that 3 to 5-year-olds discriminate real objects from toys on the basis of the domain-general notion of authenticity. Differentiating real objects from toys on the basis of authenticity is a component of children's ability to make appearance-reality distinctions. Bunce and Harris (2013) argue that the appearance-reality distinction can be based either on the ontological criterion separating real from fictional entities or authenticity, which “involves judgments about whether [an object] is genuinely what it looks like or purports to be, or is an imitation, fake, or replica.” (Bunce and Harris, 2013; p. 1494). Three-year-olds are adept at making the authenticity distinction. When projecting from animates, children could reject a toy animal knowing that the toy animal is not authentic, rather than out of the expectation that internal commonalities do not extend beyond the domain of animates. Jipson and Gelman (2007) avoided this interpretative ambiguity. They asked children to project novel features from an animal (a dog and a cat) or an artifact (a radio and a computer) to a stuffed animal. Four-year-olds consistently projected from an animal, while 5-year-olds were undecided. Children's inferences crossed the ontological boundary despite Jipson and Gelman (2007) provided clear cues to animacy by presenting movement information on videos of target objects. However, Jipson and Gelman's (2007) triad induction methodology did not give participants a positive ontological option, because a piece of electronics is just as unlikely as an animal to share properties with a stuffed animal.

Overall, projections to toys seem to be a promising avenue of research, but so far the findings are sparse and conflicting. An early knowledge view could be tested with projections involving toys, but it should be tested with a triad induction methodology that gives children a positive ontological alternative, a projection from a real category member to either a similar toy or a dissimilar category member. In order to avoid the authenticity argument based on Bunce and Harris' (2013) findings, projections within animates should be compared with projections within artifacts, for which real-toy distinction is not ontological.

### Labels and explicit category information

Labels play an important role as cues to category membership (Gelman, 2003). However, their role in inferences is not always clear (Sloutsky and Fisher, 2004). Gelman and O'Reilly, 1988 results suggest that providing superordinate category labels did not facilitate superordinate inferences. However, it is difficult to estimate the role of labels when both label and no-label condition yielded negative results. In Gelman and Davidson's (2013) superordinate induction probes, labels were presented as indicators of common category membership. Moreover, Gelman and Davidson highlighted common category membership through explicit category information. Sloutsky et al. (2015) argued that conceptual information provided by Gelman and Davidson (2013) was confused with attention-directing information (focusing children's attention on distinguishing features). In their study, Sloutsky et al. (2015) disentangled conceptual and attentional factors to show that only the latter affect young children's inferences. In order to address Sloutsky et al.'s (2015) claim that children's category-consistent responses in induction studies (e.g., Gelman and Davidson, 2013) can be explained by reference to attentional processes, it is necessary to show whether children can draw on ontological distinctions when the procedure or instruction do not explicitly draw children's attention to properties that mark the ontological boundary.

### The role of experience

Inductive inferences depend on prior experience. Children who raise fish show a different pattern of inferences within aquatic animals than children with no such experience (Inagaki, 1990). Anthropocentric bias in inductions characteristic of urban majority culture children is absent in children who have rich experience with nature (Ross et al., 2003). Children of biology experts but not children of laypeople can differentiate between real animals and toy animals when generalizing internal properties (Tarlowski, 2006). Shafto et al. (2007) argue that prior experience influences availability of features and categories during induction. Fisher et al. (2015) PaRS model posits that experience affects the outcome of similarity computation by modifying the set of representational features that enter the computation. Although the present study is not aimed to test individual differences in experience, it must be noted that any model of the development of inductive inference should account for them.

### The present study

As can be seen from this review, the role of conceptual information in induction is a highly complex, multidimensional phenomenon. Seemingly conflicting results often arise because studies employ a different combination of values on each dimension. Moreover, although each dimension received considerable attention, we are still far from having a complete picture. The present study is an attempt to explore a combination of conditions relevant to children's inductive inferences that jointly received little attention. The key distinguishing features of the present design are that it probes inferences at a superordinate level that either cross or do not cross the ontological boundary, uses a modified triad induction probe, tests inferences involving toys, relies on realistic representations (photographs) of existing objects, and presents category distinctions implicitly, by merely selecting objects from different categories, without providing labels or category descriptions. The principal motivation behind selecting this particular set of properties was to test the relatively unexplored, yet highly important issue of children's reliance on ontological constraints in induction, with the use of materials and design that provide a high level of ecological validity without compromising the control of perceptual similarity.

## Study 1

The aim of the present study was to address unresolved theoretical questions concerning the role of ontological constraints in inductive inference: Can children ignore perceptual similarity to avoid crossing the ontological boundary if this boundary is not explicitly highlighted in the design? Do ontological boundary limits constrain their inferences, such that they expect properties of real animals not to extend to toy animals while not holding an analogous expectation for the properties of real and toy vehicles? In order to address these questions, I presented children with a computerized induction task in which they projected internal properties from real dogs to either dissimilar real animals or to similar toy animals and from real cars to either dissimilar real vehicles or to similar toy vehicles. The objects were presented as color photographs and never named. Thus the category distinction was implicit. The distinction between real animals and toy animals coincides with important ontological boundary, while the distinction between real vehicles and toy vehicles does not. The early knowledge view suggests that children expect fundamental differences between ontological domains. Gelman and Davidson (2013) show that children's inferences do not cross ontological boundary separating animals from artifacts even if the perceptual similarity of the distracter is compelling. Keil (1989) showed that children do not accept perceptual transformations that cross the living-nonliving ontological boundary. If children are able to rely on ontological constraints in induction, they should make projections from dogs to real animals rather than toy animals. At the same time, they should not display a preference for real vehicles when making inferences from cars because real vehicles and toy vehicles belong to the same ontological domain of artifacts so naïve ontology does not prohibit crossing the boundary between them in inferences.

The selection of animals and vehicles as comparison items needs to be justified. The choice of animals is quite natural. It is the most salient, the best known superordinate distinction for biological beings. It has also received most attention form research. Selecting a superordinate category within artifacts was much more challenging. I chose vehicles because in many ways they are analogous to animals. They are a relatively coherent category, characterized by movement. Internal divisions within vehicles that are analogous to those within animals. Both animals and vehicles live or operate either on land, in the water or in the air. Many vehicles are designed with an eye on animate adaptations to movement. Finally, real object—toy distinction for vehicles is probably more clear and salient than for any other artifact category. Toy vehicles are ubiquitous, and for the most part, there is no doubt whether something is a real vehicle or a toy. In other artifact categories, e.g., tools, the real-toy boundary is much less apparent, mostly due to the relative lack of complexity of the real objects (consider the difference between a real and toy screwdriver).

### Method

#### Participants

Twenty-five children with mean age of 5;5, range between 5;0 and 5;10 participated in the study. Fourteen of them were girls. Children came from a small town in central Poland. Children's parents were informed about the purpose of the study and signed an informed consent form. Children provided verbal consent for participation in the study. Sixteen adults (mean age 37 years, range 24–49) provided similarity ratings for the items presented in the induction study.

#### Materials and procedure

##### Introduction to the induction study

The procedure started with an introductory activity which was a real-life analog to the experimental induction task. In this activity, children had a tangible experience of detecting internal, unobservable properties of objects.

First, the experimenter asked the children what was inside a fridge, a purse, and a human. The children readily provided answers that included food, money, and internal organs. The experimenter asked what needs to be done to see what is inside the discussed objects. The children overwhelmingly responded that the contents of the fridge could be examined by opening, while the insides of a human cannot be readily viewed in this way. The experimenter explained to the child that whenever insides of an object cannot be viewed, people can use detectors, devices that signal the presence of invisible internal properties. The experimenter taught the child how to use a toy metal detector to find a metal plaque inside one of two envelopes. The child was presented with a pair of envelopes and had to use the detector to find the metal plaque. Only one of the envelopes contained the plaque. The task was a real-life analog to the computerized experimental task. After learning how to operate the metal detector the child was introduced to the concept of particles–tiny, invisible bits that can be found in all objects. At that point, the experimental procedure on a tablet began.

##### Induction task

The induction task was meant to be analogous to the triad induction task with one base item and two target items (e.g., “A dog has blicks inside. Does a teddy bear or an ant have blicks inside like the dog?”). However, there were two important differences, which I explain below.

The first difference involved the way the premise was presented. While in most induction tasks the property of the base is explicitly taught by the experimenter, in the present task it was presented implicitly. Children obtained information about the property of the base from a training trial, a trial which first presented the base along with an ontologically unrelated object, then elicited the child's selection of one of these objects, and finally offered feedback indicating that the base possesses the feature. Thus, instead of being explicitly told that a dog has blicks, a child was shown a dog and a cloud, asked to indicate which one has blicks, and received feedback pointing to the dog as the correct answer. From the methodological viewpoint, performing a training trial was equivalent to being told that the base has the feature. From the child's perspective, the teaching event became similar to the testing event, that is, the test trial. The test trial probed projections from the base to one of two target objects. Like the training trial, it presented two objects (targets) and elicited the child's selection. However, unlike the training trial, the test trial did not offer feedback.

The second difference involved the relationship between trials. Most traditional triad induction tasks involve a series of unrelated trials, that is, a child is taught a different property on each trial. In the present study, all the trials involved the same property. Thus, instead of having a series of teach A-test A, teach B-test B…sequences, the present study had a series of train A-test A, train A-test A…sequences. The training trials were interspersed with the test trials to continuously remind participants about the premise. From the viewpoint of participant experience, rather than being a series of separate problems to solve, the task was a single, highly engaging discovery activity.

At the beginning of the induction task, the experimenter entered the child's data into the program and showed the child the screen with pictures of 3 particles. The child was told that these particles are in fact so tiny that one cannot see them and that she would look for them in different objects with the use of a detector. The child was asked to pick one of the particles by touching it. Each of the tasks started with the child picking a particle. On the touch, the program played a recording “These particles are called [blicks]. I wonder which things have [blicks] inside.” The experimenter then prompted the child to touch on a vertical line that appeared in the middle of the screen. The line appeared before every trial. Its position ensured that the child's hand was precisely in-between the two objects when they appeared on the screen.

There were three separate tasks. Each one was about finding a different kind of particle. The first task served as a warm-up, its only goal was to familiarize the child with the game, and it was not a subject of any analyses. The remaining tasks were experimental and presented the *animals* condition and the *vehicles* condition in a counterbalanced order.

The warm-up task consisted of a series of seven train-test sequences, adding to a total of 14 trials. All the trials, both training and test, presented a shoe (or shoes) and a piece of furniture (a table, a chair, or a bookshelf). Each experimental task was made up of 12 train-test sequences, totaling 24 trials. In the animals task, training trials presented a dog and water (icicles, ice, clouds), while test trials presented an animal dissimilar to a dog (a category match) and a toy animal similar to a dog. In the vehicles task, training trials presented a car and a stone (or pebbles), while test trials presented a vehicle dissimilar to a car (a category match) and a toy vehicle similar to a car. The list of categories can be found in Table 1 and a full list of items in Appendix 1.

**Table 1:** Object kinds presented during the induction task in Studies 1 and 2.

**Type of task**	**Objects with the feature^a^**	**Objects with no feature^a^**
**WARM UP**		
7 training (feedback) trials	Shoe^b^/Chocolate^c^	Furniture^b^/Bread^c^
7 test (no feedback) trials	Shoe^b^/Chocolate^c^	Furniture^b^/Bread^c^
**ANIMALS**		
12 training (feedback) trials	Dogs	Water
12 test (no feedback) trials	Dissimilar animals	Similar toy animals
**VEHICLES**		
12 training (feedback) trials	Cars	Stones
12 test (no feedback) trials	Dissimilar vehicles	Similar toy vehicles

a *After the child made the response, the presence of the feature in the object was signaled on training trials only*.

b *Items presented in Study 1*.

c *Items presented in Study 2*.

The first trial was always a training trial, while the second was always a test trial. For the remaining train-test sequences the order was established randomly, that is, for some train-test sequences, the training trial preceded the test trial, on others, the test trial preceded the training trial. This design made the task less predictable, thus further blurring the distinction between training and test trials from the viewpoint of the participant. It also ensured that the maximum of subsequent trials of the same kind was two.

**Figure 1:**
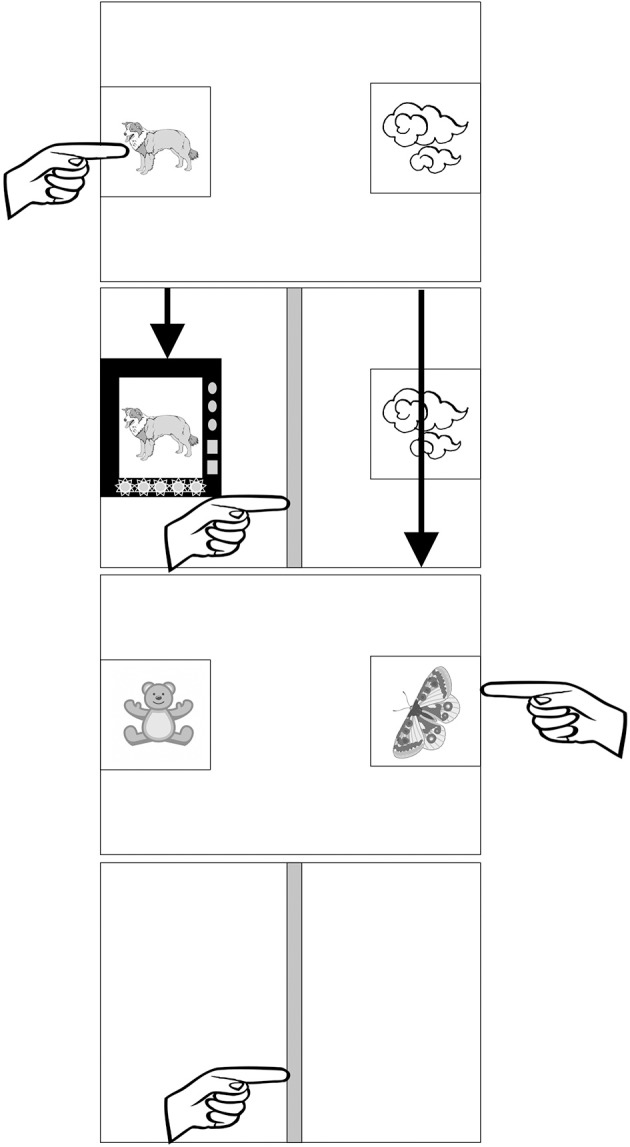
Schematic presentation of one training trial and one test trial in the induction paradigm. In the training trial, the child sees a dog and clouds and points to the dog as the object possessing the projected feature. The detectors scan the objects (movement path indicated by the arrows). One of the detectors stops over the dog. There is a beeping sound, flashing diodes, and a message “Yes! This one has blicks inside!”. The child then touches the vertical line to initiate the test trial. The test trial presents a teddy bear and a butterfly. The child points to the butterfly, both objects disappear, a vertical line appears signaling a subsequent trial.

Each task was initiated by a training trial. A pair of items appeared on the screen. The experimenter said “We have two things here. One of them has [blicks] inside, but we don't know which one, because [blicks] can't be seen. Guess what has [blicks] inside. Touch with your finger to check which one has [blicks] inside.” The objects were placed on the opposite sides of the screen, as shown in Figure 1. After the child touched on one of the objects, a detector appeared on top of each side of the screen. The detectors were represented by pictures of oscilloscopes with translucent screen parts. The detectors moved down simultaneously scanning the two objects. One of the detectors stopped and remained at the object indicating by sound and light that this object has the projected feature, while the other passed over and disappeared. When the detector “discovered” the feature it stopped in such a position that the object in the picture was clearly visible in the translucent screen part of the device. When the child selected the appropriate object, the recording said “Yes! This one has [blicks] inside!,” when the child made an incorrect choice, it said “No! The other one has [blicks] inside!” Once the recording ended, the vertical line appeared. The objects and the detector that discovered the particle remained visible until the child touched on the vertical line, thus initiating a subsequent trial, which was a test trial.

As in the training trial, the test trial started with the appearance of two items, and the experimenter's prompt “And now which one has [blicks] inside?” When the child made a selection, the objects disappeared, there was no recording, and the vertical line immediately appeared on a blank screen. Although there was no feedback from the program, warm-up test trials received feedback from the experimenter, who said: “Look, this time the detector did not show us, which one has [blicks] inside, but I think you chose the right one/ it was the other one.” The reason why the experimenter provided feedback verbally on test trials in the warm-up task was to make sure that the child did not interpret the lack of feedback from the program as an indication of an incorrect response. Importantly, no form of feedback was ever given on test trials in either animals or vehicles task.

After the first training—test sequence, there were additional 6 training—test sequences in the warm-up, and 11 in the experimental tasks. On those trials, the experimenter prompted the child by saying “And now, what has [blicks] inside? Touch with your finger.” or did not say anything if the child completed the trials without hesitation. The experimenter also provided verbal feedback on the remaining test trials within warm-up and said “The detector didn't show us, we will see at the end” on the remaining experimental test trials. See Figure 1 for a schematic depiction of an example training and test trial in the vehicles task. After the child completed all the trials within the task, the program presented a set of small circles. The number of circles corresponded to the number of correct responses the child made. The child was praised by the recording (“Look how many you've found!”) and the experimenter.

The study was administered on a 9.7-inch tablet. The objects were presented as naturalistic pictures, 5.7 by 5.7 cm. The objects were presented in 8 different random orders. The side of presentation of each object was randomized individually for each trial. Detailed instruction to the task is presented in Appendix 2.

##### Adult similarity judgments

**Figure 2:**
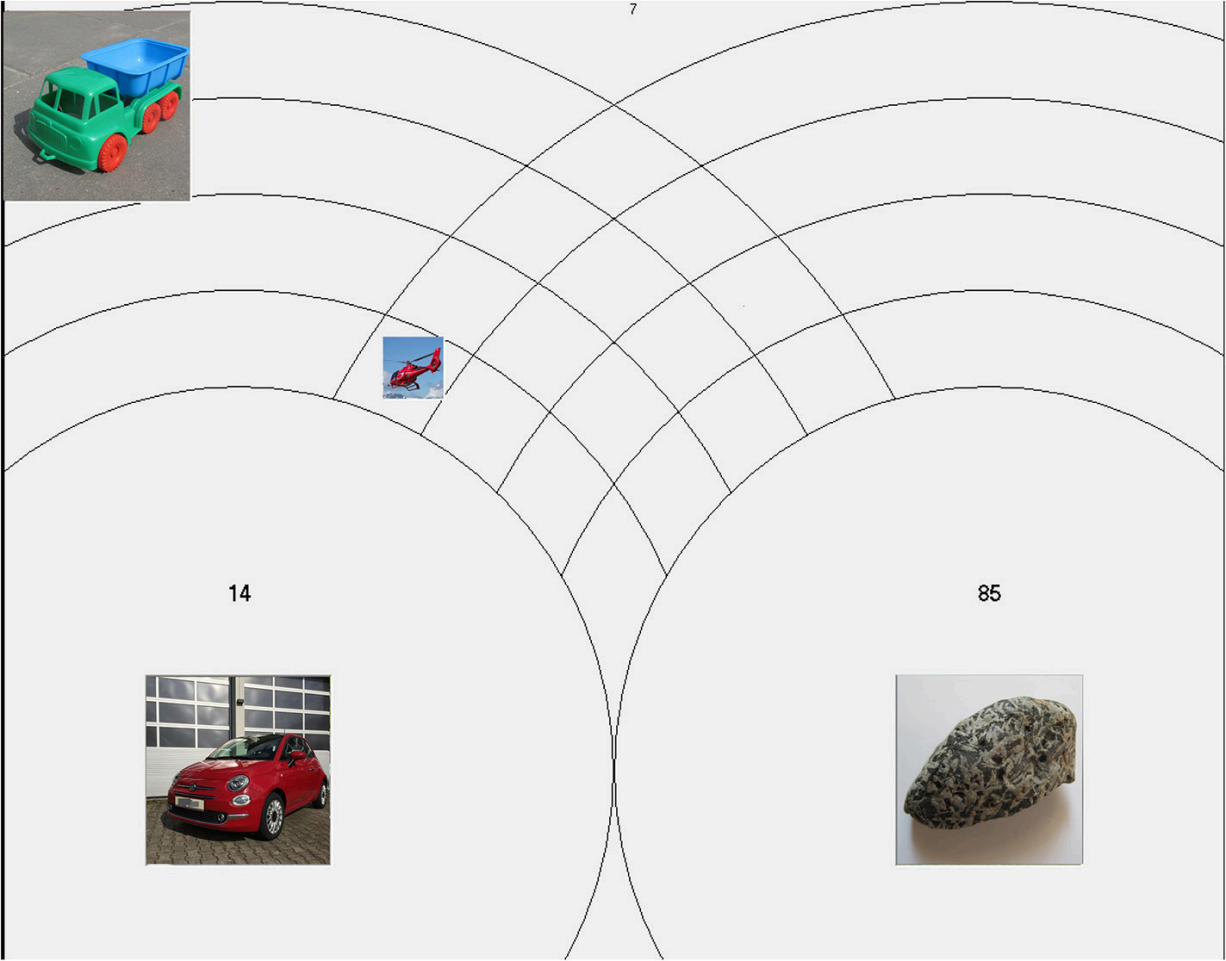
The display used in adult similarity judgment task. In this image, the photos used in the study were replaced with analogous ones due to copyright restrictions.

In order to ensure that any potential domain effects in children's induction task would not stem from systematic differences in perceptual similarity between the bases (dogs and cars) and test items (toys and real category members), I asked adult judges to assess the perceptual distance between objects presented in the children's task. The similarity judgment task was designed to resemble the induction task closely. The induction task presented a series of pairs of objects, for example, car^1^ and rock^1^, followed by a helicopter^2^ and a toy truck^2^, followed by car^3^ and rock^3^ (index representing the pair number). It is reasonable to assume that the similarity judgments on which children relied would be made between subsequent trials, that is helicopter^2^ and toy truck^2^ would be simultaneously compared to a previous pair, that is car^1^ and rock^1^. That is why the judges assessed the perceptual similarity between objects from the two subsequent pairs. For a series Pair^1^, Pair^2^, Pair^3^,…judges assessed the similarity of Pair^2^ objects to Pair^1^ objects then Pair^3^ objects to Pair^2^ objects and so on. The objects representing the previous (or reference) pair, e.g., Pair^1^ were visible at the bottom of the screen, each at the center of four circles (see Figure 2), while the objects representing a subsequent pair, e.g., Pair^2^ were visible at the top corners of the screen. Participants' task was to assess the similarity of Pair^2^ objects to Pair^1^ objects. Please note, that the assessment involves four objects, which means that there are four similarity judgments involved (helicopter^2^ to car^1^, toy truck^2^ to car^1^ and helicopter^2^ to rock^1^, toy truck^2^ to rock^1^). Participants placed each of the Pair^2^ objects in the position that reflected its similarity to objects from Pair^1^. The similarity was represented as a physical distance on a plain, so it was possible to simultaneously mark the similarity of each assessed object to the two reference objects from the Pair^1^ (for example, the position of helicopter^2^ could be described as its distance from rock^1^ and car^1^). The scale was both spatial and numerical, and it ranged from 0 (close by) to 100 (far away), where 0 meant no perceptual distance (maximum similarity) and 100 meant maximum perceptual distance (no similarity). In order to make the assessment easier, the reference objects (Pair^1^ in this example) were encircled by four rings. Spatially, the first ring corresponded to values between 0 and 25, the next corresponded to values between 25 and 50 and so on. In Figure 2, the similarity of the helicopter^2^ to the car^1^ has been assessed at 14 and the similarity of helicopter^2^ to rock^1^ has been assessed at 85. The helicopter^2^ was placed in the most inner circle for the car^1^ and the outermost circle for the rock^1^. The toy truck^2^ has not yet been assessed and it can be visible in its original position and size at the top of the screen. I decided to use this novel, simultaneous assessment of similarity task because similarity judgments are highly affected by context and children's similarity judgments in the induction task would also be simultaneous and sequential.

### Results

#### Adult similarity judgments

The similarity judgments for key comparison pairs were evaluated, namely those between the base and the toy and between the base and the real object. Each participant's perceptual distance judgments were averaged for all comparisons within each comparison type and entered into an ANOVA with domain (animals vs. vehicles) and type of comparison (base—toy vs. base—real object). See Table 2 for adults' perceptual distance judgments. There was an effect of domain *F*_(1, 15)_ = 6.19, *p* = 0.025, partial eta2 = 0.29. Overall, the similarity was greater within vehicles than within animals. There was also an effect of type of comparison, *F*_(1, 15)_ = 54.82, *p* < 0.001, partial eta2 = 0.79. The perceptual similarity between bases and toys was greater than the perceptual similarity between bases and real objects. Importantly, there was no interaction, *F*_(1, 15)_ = 0.4, which means that the distributions of similarities between bases and the two alternatives in test trials were equal in the two domains. Perceptual similarity relationships favored selecting toys equally for vehicles and animals.

**Table 2:** Adult perceptual distance judgments.

**Type of pair**	**Perceptual distance**
Car – real vehicle	85 (10.1)
Car – toy vehicle	70 (11.2)
Dog – real animal	88 (9.0)
Dog – toy animal	75 (15.9)

*The scale spanned from 0 to 100, with 0 meaning no perceptual distance (maximum similarity) and 100 meaning maximum perceptual distance (no similarity). SD in parentheses*.

#### Children's inductive inferences training trials

At the start, children did not receive any cues as to which object possesses the feature. On the first training trial, they had to make a guess. Because all training trials received feedback, there was a clear indication as to what the correct answer is on all remaining training trials. Children were correct on 0.92 training trials in animals condition and 0.95 trials in artifacts condition. The difference between conditions was not statistically significant. Only two children did not reach 8 out of 11 correct choices on training trials. This shows that children were attentive throughout the task and understood its goal. Children who did not reach the training trail correctness criterion were not included in the analysis.

#### Test trials

**Figure 3:**
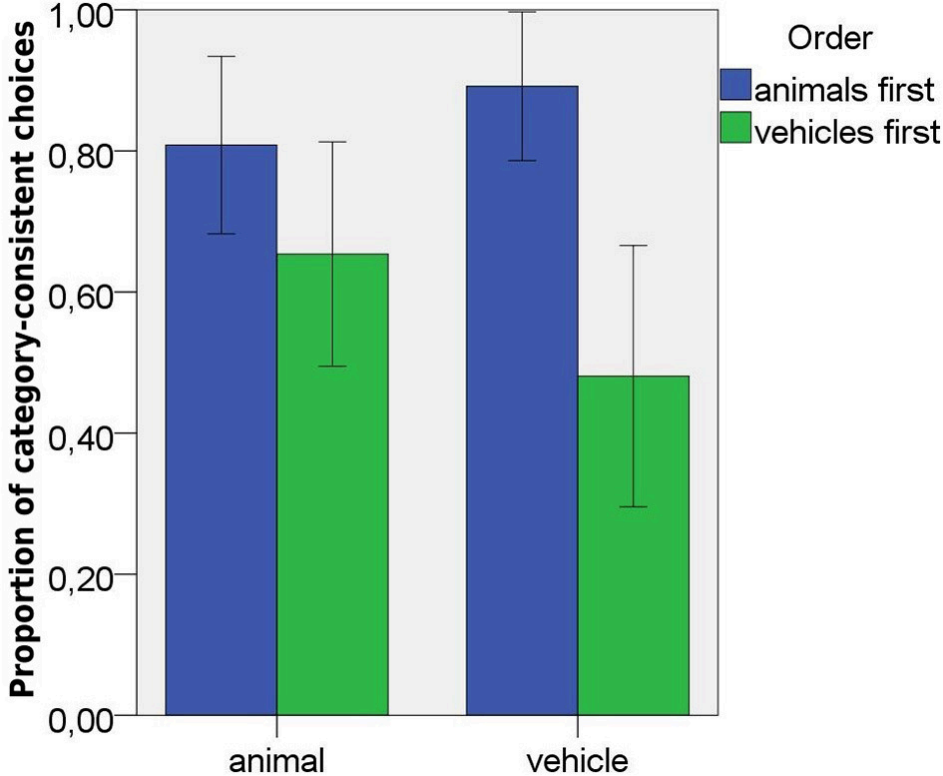
Proportions of category-consistent responses in Study 1, by domain and order of presentation. Error bars represent 95% confidence intervals.

The proportions of category-consistent choices on test trials were entered into an ANOVA with domain (vehicles vs. animals) as within-subjects variable and order of presentation (vehicles first vs. animals first) as a between-subjects variable. The analysis did not yield an effect of domain. The category-consistent selections were significantly above chance for both animals *t*_(22)_ = 4.46, *p* < 0.001 (*M* = 0.72, *SD* = 0.24) and vehicles, *t*_(22)_ = 2.38, *p* = 0.027 (*M* = 0.66, *SD* = 0.32). There was an effect of order of presentation, *F*_(1,21)_ = 9.40, *p* = 0.006, partial eta2 = 0.31. There were more category-consistent choices overall when animals were presented first (*M* = 0.85, *SD* = 0.16) than when vehicles were presented first (*M* = 0.57, *SD* = 0.26). There was a significant interaction between domain and order of presentation, *F*_(1, 21)_ = 9.45, *p* = 0.006, partial eta2 = 0.31 (see Figure 3). *Post hoc* analyses revealed that order of presentation mattered only for vehicles, *t*_(18)_ = 4.24, *p* < 0.001. There were fewer category consistent selections for vehicles when they were presented first (*M* = 0.48, *SD* = 0.31), then when they were presented after animals (*M* = 0.89, *SD* = 0.14). For animals, the order of presentation had no significant effect (*M* = 0.81, *SD* = 0.18, when presented first and *M* = 0.65, *SD* = 0.26, when presented second). When only the first experimental tasks were compared, children made more category-consistent projections for animals (*M* = 0.81, *SD* = 0.18) than for vehicles (*M* = 0.48, *SD* = 0.31), *t*_(20)_ = 3.22, *p* = 0.004. Within-subjects comparisons showed that children who viewed artifacts first made fewer category-consistent projections to artifacts than to animals, *t*_(12)_ = 2.44, *p* < 0.031. Interestingly, children who viewed animals first made fewer category-consistent projections to animals than to artifacts, *t*_(9)_ = 3.87, *p* = 0.004. This significant effect reflects a very small difference in proportions (0.81 vs. 0.89 category-consistent choices) and is related to the fact that for 7 out of 10 children, the number of category-consistent responses increased and for 3 it remained the same.

### Discussion

The results showed an influence of conceptual information on inductive inference. Children who started with the animals task preferred category-consistent responses and they continued to make category-consistent responses in the vehicles task. Children who started with the vehicles task made mixed responses, but they shifted to category-consistent responses in the animals task. There were two categorical distinctions that could potentially inform category-consistent selections in this paradigm. One was the authenticity distinction, and the other one was the ontological distinction. While the first applied equally to both conditions, the second was limited to the animal condition. If children relied on authenticity by default, their first response would be to reject toys both in the animal and vehicle condition. However, consistent rejection of toys only occurred in the animal condition. This suggests that, in their first response to animals, children observed the animate ontological boundary limit rather than authenticity. The fact that children who rejected toy animals on the first task went on to reject toy vehicles on the second task suggests that ontologically driven response in the animals task brought the authenticity distinction to the foreground in the vehicles task.

The comparison between the first tasks suggests that, by default, children's inductions rely on ontological constraints and not authenticity. However, the order effect for vehicles shows that children can make category-consistent inductions within artifacts as well. The fact that children made mixed responses to vehicles presented first and category-consistent responses to vehicles presented after animals is worth special attention. If responses to vehicles followed the same pattern of few category-consistent choices regardless of the order of presentation, it could be argued that the greater reliance on category-consistent choices in the animals than vehicles condition is a result of between-domain differences in the processing of perceptual similarity. Although adult data suggests that perceptual similarity relations in the animals and vehicles condition were equal, it could be possible that children process perceptual similarity differently than adults do, and that their impression of perceptual similarity only favors category-consistent choices in the animals condition. Category-consistent responding in vehicles task presented second invalidates such interpretation, and it highlights the role of experimentally induced conceptual or attentional factors in inductive inferences. It shows that children's experience with the animals task induced children to view the artifacts in the subsequent task through the lens of real-toy distinction.

The observed pattern of responses cannot be due to children's attention being drawn to a single perceptual feature as in Gelman and Davidson (2013). Please note that toys were made of diverse materials. Most toy animals were plush to ensure high perceptual similarity to dogs. Vehicle toys were made of wood, metal, and plastic. No readily available and constant set of perceptual features marks the toy-real distinction in the sets presented in the study. The toy-real distinction necessarily relied on category knowledge rather than immediate perception. This suggests that the order difference in how children made inductions within vehicles is due to conceptual knowledge made salient by the animals task.

In sum, the results suggest that children are guided by an intuition that internal commonalities spanning animals do not extend to nonliving things, even if they are highly similar. Children pick up on subtle markers of animacy in photographs, and do not require information about movement, explicit category information or labels to overcome the conflicting perceptual information. The findings seem to support the early knowledge view. However, they could also be explained by the PaRS model. Children could favor category-consistent responses for animals and not for vehicles if their conceptual representations of features common to real animals and differentiating them from toy animals were richer and more easily accessed than the corresponding representations within vehicles. This account only invokes conceptual features and does not invoke category knowledge.

Although the present results seem to favor the role of conceptual information in induction, they do face a serious challenge. Adults' similarity judgments suggest that similarity relationships within the triads did not vary across domains. However, it cannot be ruled out that children perceived the similarities differently than the adults and their computations of perceptual similarity favored category-consistent choices for animates. Moreover, it is possible that the between domain differences are related to the diversity of experiences or interest in tested items. It is reasonable to expect that boys show more interest in vehicles than girls do. They may also have more experience playing with toy vehicles. However, comparisons across genders were not possible in Study 1 due to small sample size.

## Study 2

In order to address the problems with Study 1, I carried out Study 2 with an additional condition in which children, rather than making inductive inferences, assessed similarities. The similarity task will allow to confirm similarity data obtained from adults.

The warm-up task in Study 1 employed shoes and furniture, which, although unrelated to vehicles, are artifacts nonetheless. To avoid any bias from the warm-up I replaced shoes and furniture with chocolate and bread. Foodstuffs are in-between natural kinds and artifacts so their ontological status should not bias responses in either target domain.

The induction condition in Study 2 was an exact replication of Study 1 with a small change in the selection of items for the warm-up task (see Table 1). Additionally, a separate group of children completed the similarity condition. In a task strictly analogous to the induction task, children were asked to make similarity assessments. In addition to replicating Study 1 results in induction condition, I expected that the similarity task would yield more similarity-based choices than category-consistent choices. I also expected no domain differences in similarity judgments. Small sample size in Study 1 made it impossible to analyze the role of gender. Sample size in Study 2 was increased which made it possible to enter gender into the analyses.

### Method

#### Participants

Eighty-one children participated in the study, with a mean age of 5;3 (ranging between 4;1 and 6;2), 38 of them were girls. One child did not complete the whole procedure and thus was excluded. Five additional children were excluded on the basis of training trial performance (see section Results). Of the remaining 75 children, 38 were assigned to the induction task and 37 to the similarity task. Children attended four different preschools, either from a small town or a large city in central Poland. Children were tested by three different experimenters, including the author. Children's parents were informed about the purpose of the study and signed an informed consent form. Children provided verbal consent for participation in the study.

#### Materials

Exactly the same set of items was used in the experimental tasks. However, the items in the warm-up task were changed (see Table 1). As in the previous study, children's performance in the warm-up was not analyzed.

#### Procedure

The procedure of the induction task was exactly the same as in Study 1. The similarity task was designed to maximize structural analogies to the induction task. In the introduction to the similarity task, the experimenter first drew the child's attention to the fact that some things look alike while others are different. The experimenter showed the child a picture frame and said that they would place similar looking things in the frame. She then placed a candle in the frame and produced a carrot and a coconut, asking the child to put the thing that is similar to the candle in the frame. This task was repeated with an analogous set of objects. After that, the experimenter showed the child an envelope and said that there was a mystery object in it. She put the envelope under the frame. She then produced two objects and said that one of them was similar to the mystery object. The child had to guess which one it was. The objects were a piece of red-white cloth and a piece of cardboard. After picking an object, the child was instructed that the piece of cloth was similar to the mystery object. Two more pairs of objects were presented to the child, one of which was a similar-looking piece of cloth, while the other was a dissimilar piece of a different material. The child was expected to select the piece of cloth on two consecutive trials and was always given feedback. If she did not make the correct choice, the initial trials were repeated until 3 consecutive hits. All the children succeeded in this task. In the end, the experimenter showed the child the mystery object, which turned out to be a piece of red-white cloth.

After this introduction, the experimenter said that they would play the same game on the tablet. The program was the same as for the induction task, with the exception that instead of pictures of particles in the initial panel, there were three frames with question marks representing three mystery objects. The child was told that she would be trying to find things that were similar to the mystery objects. Once the child selected the mystery object, the object was introduced by a play name—blick, dax or tul, through a recording.

The child was presented with the same set of tasks: warm-up, animals, and vehicles. The design of the tasks was the same as in the induction condition except that on feedback trials, frames appeared instead of detectors and the voice said “Yes! This one is similar to [the blick]!” or “No! The other one is similar to [the blick]!.”

### Results

#### Training trials

Children's correct responses reached a mean of 0.97 (*SD* = 0.10) for vehicles and 0.95 (*SD* = 0.12) for animals in the induction task and 0.96 (*SD* = 0.06) for vehicles and 0.95 (*SD* = 0.11) for animals in the similarity task. These values did not significantly differ. Five children did not reach the 8 out of 11 correctness threshold for responses on at least one of the tasks and were thus excluded from the analyses. The training trials data shows that the children were predominantly attentive and understood the purpose of the task.

#### Test trials

**Figure 4:**
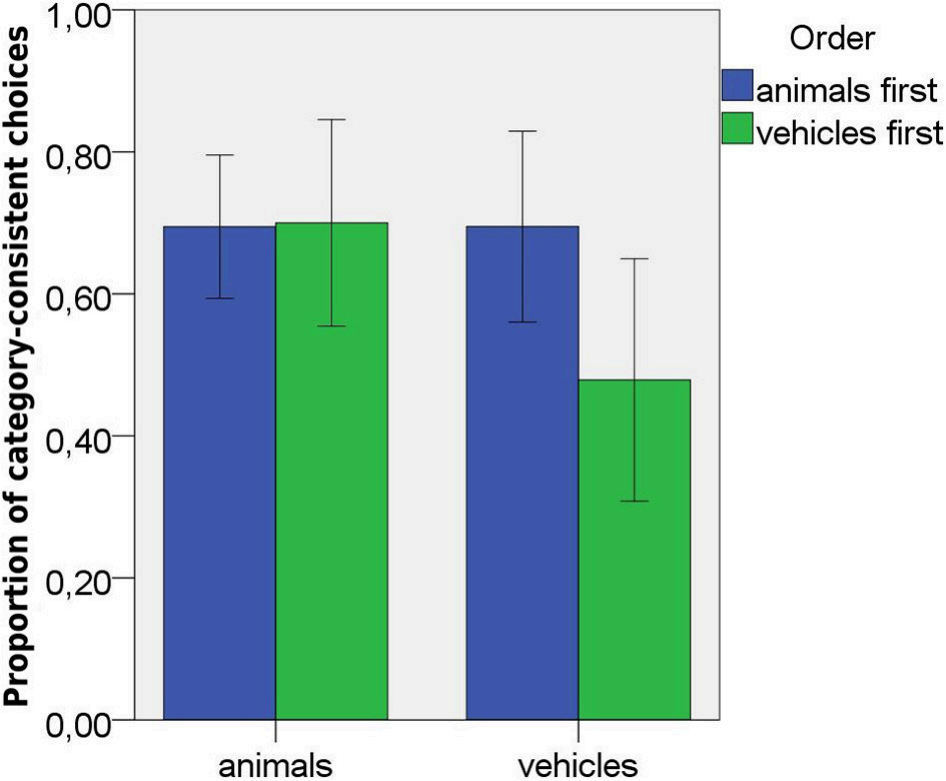
Proportions of category-consistent choices in Study 2 induction task as a function of domain and order of presentation. Error bars represent 95% confidence intervals.

**Figure 5:**
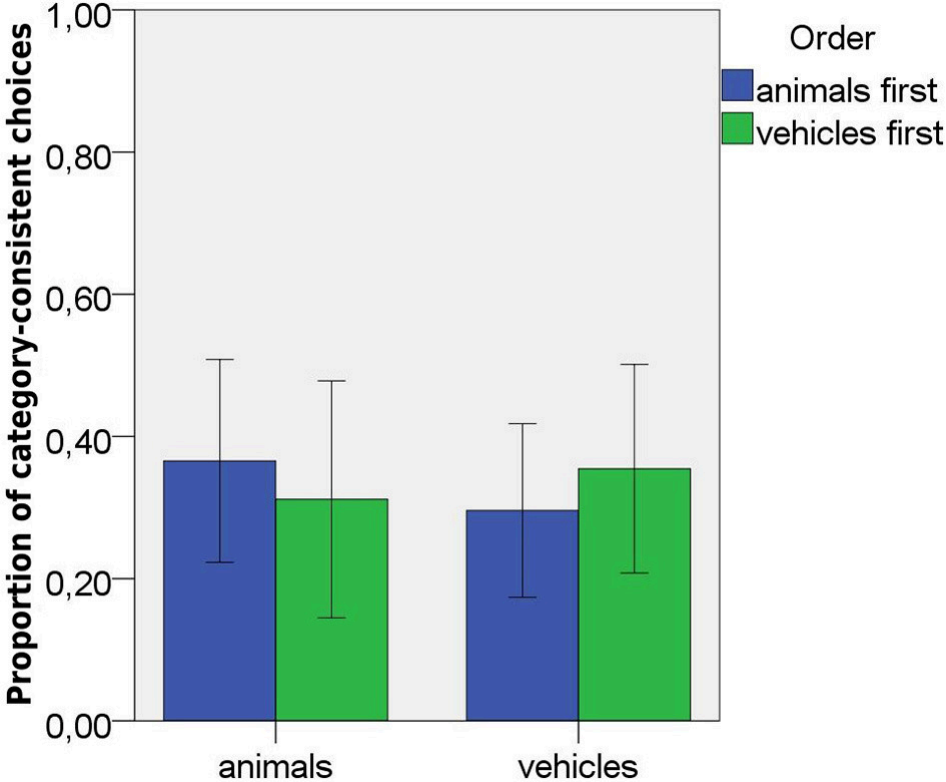
Proportions of category-consistent choices in Study 2 similarity task as a function of domain and order of presentation. Error bars represent 95% confidence intervals.

I entered the proportions of category-consistent choices in the test trials into an analysis of variance with domain (vehicles vs. animals) as within-subjects factor and type of task (similarity vs. induction), order of presentation (vehicles vs. animals first), and gender as between-subjects factors. The analysis revealed an effect of type of task, *F*_(1, 67)_ = 26.45, *p* < 0.001, partial eta2 = 0.28. Children made more category-consistent selections in the induction (*M* = 0.64, *SD* = 0.27) than in the similarity task (*M* = 0.33, *SD* = 0.26). There was a marginal effect of domain, *F*_(1, 67)_ = 3.55, *p* = 0.064, partial eta2 = 0.05. Children made more category-consistent selections for animals (*M* = 0.52, *SD* = 0.34) than for vehicles (*M* = 0.46, *SD* = 0.33). There was no domain by task interaction, *F*_(1, 67)_ = 2.36, NS. There was, however, and interaction between domain, task, and order of presentation, *F*_(1, 67)_ = 6.86, *p* = 0.011, partial eta2 = 0.09 (see Figures 4, 5 and Table 3). *Post hoc* comparisons showed that animals did not differ from vehicles in the similarity task regardless of the order of presentation, both ts < 1, but in the induction task, vehicles received fewer category-consistent responses than animals when they were presented first, *t*_(19)_ = 3.16, *p* = 0.005. When animals were presented first, there was no domain difference, *t* < 1. Comparisons between induction and similarity tasks revealed significant differences for animals in both orders, and for vehicles presented second (all ts > 3). In all these conditions there were more category-consistent selections in the induction task. There was no difference between similarity and induction task for vehicles presented first, *t*_(37)_ = 1.15, NS. Order of presentation mattered for vehicles in the induction task. They received fever category consistent inferences when presented first then when presented second, *t*_(36)_ = 2.05, *p* = 0.047. It did not matter for animals in the induction or for either domain in the similarity task, all ts < 1. Finally, when the first tasks were compared, there was a significant difference between animals and vehicles in the induction task *t*_(30.3)_ = 2.28, *p* = 0.03 (0.69 vs. 0.48 category-consistent choices), but not in the similarity task (0.37 vs. 0.35 category-consistent choices).

**Table 3:** Proportions of category-consistent selections in Study 2 by task, domain, order of presentation and gender.

**Task and gender**	***N***	**Animals**	**Vehicles**	**Total**
**INDUCTION**				
**Animals first**				
Boys	10	0.73 (0.21)	0.68 (0.30)	0.71 (0.24)
Girls	8	0.65 (0.19)	0.71 (0.25)	0.68 (0.20)
Total	18	0.69 (0.20)	0.69 (0.27)	0.69 (0.22)
**Vehicles first**				
Boys	10	0.62 (0.37)	0.29 (0.31)	0.45 (0.29)
Girls	10	0.78 (0.23)	0.67 (0.32)	0.72 (0.25)
Total	20	0.70 (0.31)	0.48 (0.36)	0.59 (0.30)
Total	38	0.70 (0.26)	0.58 (0.34)	0.64 (0.27)
**SIMILARITY**				
**Animals first**				
Boys	10	0.42 (0.26)	0.28 (0.20)	0.35 (0.21)
Girls	8	0.30 (0.33)	0.31 (0.31)	0.31 (0.21)
Total	18	0.37 (0.29)	0.30 (0.25)	0.33 (0.21)
**Vehicles first**				
Boys	9	0.41 (0.36)	0.39 (0.31)	0.40 (0.32)
Girls	10	0.23 (0.33)	0.33 (0.31)	0.28 (0.30)
Total	19	0.31 (0.35)	0.34 (0.30)	0.33 (0.31)
Total	37	0.34 (0.32)	0.33 (0.28)	0.33 (0.26)

*Standard deviations are presented in parentheses*.

To complement these *post hoc* analyses I also compared children's performance against chance (0.5) in conditions divided by domain, task, and order of presentation. In the induction task, animals presented first were above chance, *t*_(17)_ = 4.07, *p* = 0.001, so were vehicles presented second *t*_(17)_ = 3.06, *p* = 0.007, and animals presented second, *t*_(19)_ = 2.88, *p* = 0.01, but not vehicles presented first *t*_(19)_ = −0.26 NS. In the similarity task, animals presented first and vehicles presented first were marginally below chance, *t*_(17)_ = 1.99, *p* = 0.06 and *t*_(18)_ = 2.08, *p* = 0.052 respectively. Vehicles and animals presented second were significantly below chance, *t*_(17)_ = 3.52, *p* = 0.003 and *t*_(18)_ = 2.37, *p* = 0.029, respectively.

Additionally, there was an interaction between domain and gender, *F*_(1, 67)_ = 5.37, *p* = 0.024, eta2 = 0.07 and a marginal interaction between gender and task, *F*_(1,67)_ = 2.84, *p* = 0.096, partial eta2 = 0.04. Because gender entered into interactions with key experimental variables I carried out 2 × 2 × 2 (domain by task by order of presentation) ANOVAS separately for boys and girls. The analysis for boys yielded an effect of domain, *F*_(1, 35)_ = 11.56, *p* = 0.002, partial eta2 = 0.25, and task, *F*_(1, 35)_ = 5.84, *p* = 0.021, partial eta2 = 0.14. There was also an order by domain by task interaction, *F*_(1, 35)_ = 6.26, *p* = 0.017, partial eta2 = 0.15. For boys, there was a significant difference between induction and similarity tasks for both domains, but only when animals were presented first. When vehicles were presented first, the differences did not reach significance for either animals or vehicles. In a complementary analysis for girls, the only significant effect obtained for the type of task, *F*_(1, 32)_ = 24.23, *p* < 0.001, partial eta2 = 0.43. For girls, task differences appeared in all conditions, but there were no domain differences.

### Discussion

Study 2 confirmed the findings of Study 1 by providing more evidence that children rely on conceptual information when making inductive inferences. Just as in Study 1 the results showed that children made category-consistent responses to animals regardless of the order of presentation and to vehicles when they were presented after animals, but not to vehicles presented first. At the same time, children made similarity-based choices in the similarity task in both domains, regardless of the order of presentation. Study 2 included gender as an additional variable, and the analyses showed differential patterns of responses for boys and girls. The responses of boys corresponded to the pattern described above, with category-consistent responses in all the induction probes except for vehicles presented first, and similarity-based or mixed choices in the similarity task. Girls showed a strong tendency to make category-consistent choices in the induction task and similarity-based choices in the similarity task, regardless of domain. The similarity task results reinforce the finding coming from the adult task in Study 1 suggesting that the similarity relations were equivalent between domains and that they favored toys. Adult judgments were reflected in children's responses that showed below-chance responses and no domain differences.

The results suggest that children's inferences universally map onto the the ontological distinction between real animals and toys, but they also suggest that the role of real-toy distinction in the context of vehicles may be greater than Study 1 revealed. As in Study 1, Study 2 suggests that real-toy distinction is activated by the animals task and carries to the vehicles task. But the findings also show that it can be activated spontaneously as well, as it happened with girls participating in this study. The most likely explanation lies in individual differences in experience with categories (Shafto et al., 2007; Fisher et al., 2015).

## General discussion

There are many factors affecting the reliance on categories in induction. When combined, they create a large variety of conditions, some of which remain unexplored. In contrast to extant research, the present project explored superordinate-level inferences over real objects presented on photographs, used toys as different category lures, and did not provide any verbal information about category membership. Two studies probed children's tendency to resist inferences crossing ontological and / or real vs. toy category boundary even if the distinct category alternative was more perceptually similar than the category match. In counterbalanced order, children projected an internal property of real dogs to either dissimilar real animals or similar toy animals, and an internal property of real cars to either dissimilar real vehicles or similar toy vehicles.

I hypothesized that children's inferences in these tasks could be driven by distinctions involving perceptual similarity, authenticity, and ontology. The perceptual similarity and authenticity distinction applied to both the animals and vehicles task. On the one hand, I selected bases (dogs and cars) to be perceptually more similar to toys than to category matches. On the other hand, bases and category matches were authentic objects, while toys were replicas. The ontological distinction applied to the animals task but not the vehicles task. Dogs and their category matches belonged to a single ontological domain of animals, while toy animals were artifacts. Cars, other vehicles and toy vehicles all belonged to a single ontological domain of artifacts.

The results showed that children tended to generalize internal properties within real animates. In both Study 1 and 2, regardless of the order of presentation, both boys and girls, preferred to project the novel feature from a dog to a dissimilar animal than to a similar toy. The results also showed that the tendency to constrain inferences to real vehicles was conditional on the order of presentation and gender. Children consistently projected the properties of cars to other real vehicles when the vehicles task was presented after the animals task. However, when vehicles task was presented first, the pattern of responses was more complex. In Study 1, children's projections within vehicles presented first were at chance, in Study 2 boys preferred perceptually similar toys while girls preferred real vehicles.

This pattern of results suggests that, within the studied population, children's inferences universally map onto the ontological animate-inanimate distinction, while the consistency with the authenticity distinction depends on the context and individual characteristics of a child. Children's inferences mapped onto the authenticity distinction between real vehicles and toys when the vehicles task was presented after the animals task. Additionally, Study 2 also showed that girls constrained their inferences to authentic vehicles as a default. What underlying processes are responsible for the observed pattern of inferences?

### Perceptual similarity

The most straightforward explanation of the results would be that, despite my efforts to select toy representations to be more similar to bases, children viewed category matches as more perceptually similar to the bases and thus selected them on the basis of perceptual similarity. In order to rule out this possibility, in Study 1 I asked adult participants to rate the similarity of objects presented in the study. Adult judges confirmed that toys were perceptually more similar to bases than category matches were, and this difference held in both domains. More importantly, in Study 2, I tested children's perceptual similarity judgments over the same set of items, in a procedure that was strictly analogous to the induction task. Consistent with adult data, the similarity task confirmed that children perceived bases as more perceptually similar to toys than to category matches, and this difference was constant irrespective of the domain, gender or order of presentation. Thus, the responses in the induction task, particularly when it involved animals, were entirely discordant with the responses in the similarity task.

Sloutsky et al.'s (2015) argument provides additional explanation of the present results that is consistent with the perceptual account. It could be possible that the induction task drove children's attention to a single specific feature that differentiated real objects from toy replicas. Unlike in previous studies, conceptual or categorical distinctions were not signaled by verbally presented labels or feature descriptions. Objects were presented on photographs unaccompanied by verbal description, so children were only exposed to their static perceptual features. Thus the hypothetical feature driving children's attention to real-toy distinction would have to be visible in the pictures. Could it be possible that a constant single perceptual feature or set of features marked toy-real distinction and served as a guide to induction? Toys chosen for the study were made from a variety of materials using diverse techniques, category matches represented a vast range of objects, so no directly available perceptual features were constant for any of the groupings. The distinctions between toys and real objects involved, among others, texture, relations between parts, color, and the level of detail. Those feature configurations met the criterion of relational features that Sloutsky et al. (2007) or Badger and Shapiro (2015) used in their materials (such as the ratio of buttons to fingers). The findings from their studies suggest that the reliance on relational features in induction is a late developmental achievement. It can thus be concluded that the attentional process based on a single feature or a combination of features is not a feasible explanation of category-consistent inferences in the present task.

Therefore, the data presented here strongly support an argument that when making superordinate inferences within real-life objects presented on photographs, in the absence of verbal or other attention cues guiding to category membership, children are able to ignore perceptual similarity and factor in conceptual information. This conclusion runs counter to the perceptual view and is consistent with the early knowledge view as well as the PaRS model.

### Perceptual and representational similarity

The PaRS model (Fisher et al., 2015) goes beyond the perceptual processing and posits that children's inferences rely on processing similarity over both perceptual and conceptual properties. Conceptual properties constitute memory representations of direct and indirect experiences with the objects. This richer account offers an explanation of the present results that does not invoke category or ontological knowledge. Undoubtedly, children had direct or indirect experiences with the set of objects presented in the study. They witnessed self-generated movement in animals, functional properties of vehicles, and they knew that toys afford different kinds of interactions than real objects. Category-consistent inferences may have emerged from processing similarity if the conceptual component had more weight than perceptual component.

The domain difference obtained in this study may also have resulted from children's varied experiences with animals, vehicles, and their toy representations. It must be noted here that the study did not test for between-domain differences in familiarity with the objects. It is possible that children were more familiar with properties that differentiate real animals from toy animals than with properties that differentiate real vehicles from toy vehicles. This could manifest itself in more category-consistent responses for animals. It is also possible that the familiarity with test items was in some way related to gender, thus producing gender effects in Study 2.

How could PaRS explain the order effects? The real-toy distinction for animals and vehicles involves partly overlapping sets of conceptual features. For example, a dichotomous feature “engages in self-generated motion” only differentiates real from toy animals, while “is used by children to play” differentiates all toys from real objects (at least vehicles and animals). Experience with the animals task could have highlighted those conceptual features that also apply to real-toy vehicle distinction and thus promoted category-consistent responses. This account seems parsimonious as it employs specific experiences instead of invoking abstract knowledge or domain theories with contested origins (Sloutsky, 2010).

One factor that potentially distinguishes the PaRS account from the early knowledge accounts relates to variability within individual responses. PaRS is a similarity-based model. It implies that each individual inference is made on the basis of a set of features that directly apply to the compared items. This suggests greater variability between trials than predicted by the category-based model in which responses are always made on the basis of common category membership. It must be noted that there was considerable variability in children's inductions even within animals. The fact that children selected 30% of toys suggests that they did not uniformly rely on ontological constraints. 19 out of 38 children chose at least 9 out of 12 real animals in the induction task, only one child did the opposite. The rest of the children gave mixed responses. Given this variability, it is possible that only a subset of children made choices based on a fixed ontological rule. The rest could have been making individual decisions for each item based on a computation of similarity. The present results show that Fisher et al. (2015) focus on the role of individual differences in induction is a necessary next step in the study of the nature of conceptual contributions to induction.

It remains to discuss how PaRS deals with the results of the similarity task from Study 2 showing that, regardless of domain, the order of presentation, or gender, children judged bases to look more like toys than the category matches. This suggests that, when making these judgments, children blocked the representational similarity and only relied on perceptual similarity. What criteria do children use to either block or engage representational similarity? PaRS does not propose that the features children represent about the objects are structured around a domain theory. One of the properties of reasoning based on naïve theories is that it flexibly adjusts to the specific causal structure of the situation. In people's mind, a dressed man jumping into a pool is a drunk, but if there is a drowning child in the pool, he immediately turns into a hero (Medin, 1989). Children make category-consistent projections for features that are category-relevant but not for the ones that are accidental (Gelman, 1988). Without invoking naïve theories, it is not clear what criteria children rely on to flexibly adjust the properties entering the similarity judgments that are purported to underlie all their responses.

### Authenticity

Differentiating real objects from toys on the basis of authenticity is a component of children's ability to make appearance-reality distinctions (Bunce and Harris, 2013). Responses based on the authenticity criterion fall under the scope of early knowledge views because they imply the use of a categorical distinction in inference. Is it possible to explain the present pattern of results by invoking the authenticity criterion alone? Three-year-olds are adept at making the authenticity distinction, so it was clearly available to children participating in this study (Bunce and Harris, 2013). The authenticity account has the most straightforward explanation for order effects. If the same criterion underlies category-consistent responses in both tasks, its activation in the first carries over to the second. The domain differences could be explained by children's adherence to the authenticity criterion alone, on the assumption that the dimension of authenticity is more salient in the animals task than in the vehicles task, since there were more category-consistent inferences for animals presented first than for vehicles presented first. However, the difference in salience requires an explanation. Why would the authenticity distinction be more salient for animals than for vehicles? More importantly, in Study 2 girls made more category-consistent inferences for vehicles presented first than boys did. Why would the distinction between real and toy vehicles be more salient for girls than for boys? This seems counterintuitive. After all, it can be reasonably assumed that boys show more interest in vehicles and they play with toy vehicles more than girls do. In order to answer these questions it would be necessary to separately probe children's intuitions of authenticity for the set of test items.

### Ontological constraints

The explanation based on the ontological distinction relates children's category-consistent inferences to their domain theories. Ontological intuitions partition the world into phenomena that are governed by qualitatively different sets of general causal rules. For example, according to these intuitions, animals have essences (Gelman, 2003) and intentions (Carey, 1985), while artifacts are made by humans to serve a specific function (Diesendruck et al., 2003). On this account, when projecting internal properties of dogs, children's selections of dissimilar animals were driven by an essentialist intuition according to which animals share invisible internal commonalities (Gelman, 2003). On the other hand, their rejections of similar toy animals were based on an intuitive notion that toy animals belong to a different basic category of existence than real animals. No such theory-based criteria exist to prompt category-consistent responses in the vehicles task.

How does the ontological account explain the order effects? There are two possibilities. According to Gelman (2003), rather than being a unitary phenomenon, essentialism is a combination of several more basic tendencies that include appearance-reality distinction, tracking identity over time, causal determinism, induction from property clusters, and deference to experts (Gelman, 2003; p. 314). Employing essentialist reasoning to the problem involving animals would then activate multiple components of the essentialist construal, and the appearance-reality component could then highlight the authenticity distinction in the vehicles task, thus promoting category-consistent responses. There is also a more mundane explanation of order effects that is consistent with the ontological view. The carry-over effect may have been related to what children avoided rather than what they selected. Real animals and vehicles have little in common but, in children's minds, toys are a coherent grouping. Children who started with the animals task consistently avoided toys, and they may have continued to avoid toys in the vehicles task.

### Gender differences

Gender differences obtained in the study were not expected so it is important to account for them at this point. Gender differences are limited to the vehicles task presented first, in which boys chose similar toys while girls chose within-category matches.

Boys could have made more similarity-based choices for vehicles than girls because they weigh functional information more. If this were the case, there should be gender differences in the proportion of category-consistent choices across a variety of real vehicles. Real vehicles could be divided into three functional classes based on the mode of locomotion. There were 4 boats, 4 flying, and 4 wheeled vehicles. The wheeled vehicles shared the mode of transport with cars, which means they were functionally more similar to cars than the boats and the flying vehicles. If children relied on functional similarity between items they would select the wheeled vehicles more frequently than the other vehicles. I tested this hypothesis in a type of vehicle type by task by gender ANOVA which revealed an effect of vehicle type *F*_(2,142)_ = 20,44, *p* < 0.001 partial eta2 = 0.22. As expected, post hoc analyses revealed that wheeled vehicles received more selections overall (*M* = 0.58, *SD* = 0.34) than planes (*M* = 0.41, *SD* = 0.39) and boats (*M* = 0.38, *SD* = 0.38). Importantly, there was no interaction between gender and any of the remaining variables (all Fs < 1). The wheeled vehicle advantage held both for girls and boys, and it held both for induction and similarity task. This means that functional information was relevant for both boys and girls and boys did not display a stronger tendency to differentiate category matches based on functional properties than did the girls. Also, this means that children relied on functional information both in the context of making inferences and rating similarity.

Alternatively, gender differences could result from the greater attractiveness of toy vehicles for boys. This interpretation is unlikely because there is no reason why the attractiveness effect should disappear in the vehicles task presented second. Moreover, it should also be manifested in the similarity task, with boys producing more similarity-based responses than girls. The results show it was not the case. This suggests that gender difference may not have been driven by a special status of toys in boy's experience but rather by the greater availability of toy-real vehicle distinction for girls. Note that both boys and girls relied on this distinction when making inference in vehicles task presented second. This means all the children had this distinction available, but boys did not rely on it by default when vehicles were presented first. It is likely that the girls did. Understanding why they do requires additional research probing children's familiarity with test items.

### The design of the study

One factor that sets the present study apart from past research is the use of a novel design of induction task. It is often the case that the research results are specific to the design. A bulk of studies on inductive inference rely on contrived animals (Sloutsky et al., 2007; Badger and Shapiro, 2012; Gelman and Davidson, 2013). The use of unrealistic stimuli begs the question of whether the results apply to real objects as well. The designs that test superordinate inferences are often open-ended and elicit few inferences spanning superordinates (Carey, 1985; Gelman and O'Reilly, 1988). Extending the repertoire of research methods is particularly important in studies on inference within superordinates, as the few extant studies face severe challenges regarding proper control of within and between category similarity. The present paradigm is specifically designed to test higher order distinctions. Children's responses to multiple items belonging to the same superordinate can be probed without the need to introduce several unknown features. Instead, the child discovers a single unknown property in a series of trials involving a set of objects representing the probed category. This ensures that the specifics of the probed features do not introduce additional variability in responses. The fact that the child is given feedback on 50% of trials and has to make a demanding choice on the rest of the trials contributes to sustained interest in the task. Another important distinguishing feature of the procedure is that it attributes the features to bases in a way that makes intuitive sense to children. Before being taught about internal properties, they are given a tangible experience of discovering internal proprieties in real objects (they learn to use a metal detector). Additionally, the procedure verifies whether children actually learned to attribute the internal property to the base and whether they pay attention to the task by measuring performance in training trials. Most children have no difficulty understanding and following the procedure, as evidenced by the high percentage of correct responses on training trials.

## Conclusions

The present study provides substantial evidence that children's superordinate level inferences are guided by conceptual rather than perceptual information, even when conceptual information is implicit and the stimuli are realistic. The most striking and consistent is children's ability to differentiate between animate and inanimate objects in the induction task. Past research established that children differentiate between animals and inanimates on a variety of dimensions (Gelman, 2003; Opfer and Gelman, 2011) but it was unclear whether they are able to use the implicit knowledge about commonalities within animates to make category-consistent selections in an inductive task, when faced with alternative perceptual choices. Note that the majority of past triad induction studies required children to make inferences within a basic level category, a level that has the highest inductive power (Gelman and Markman, 1986; Sloutsky et al., 2007; Badger and Shapiro, 2012). Gelman and Davidson (2013) who showed children's reliance on ontological distinctions in induction employed artificially created stimuli and highlighted category distinctions by providing labels and rich conceptual information indicating which objects are animates and which are artifacts. Their study suggests that information about animacy, when explicitly highlighted, can override perceptual similarity in induction. However, it faces criticism, because category information may affect attentional weighting of features (Sloutsky et al., 2015). The present study avoids this challenge, by showing that ontological boundaries affect inferences even when they are not highlighted in any way. In the present study, children were free to decide for themselves which aspects of the stimuli were relevant for induction. They received no verbal information about the items. Still, they consistently favored within-category responses over perceptual similarity for animates.

I presented three alternative proposals concerning the cognitive processes potentially underlying the pattern of inferences observed in the induction task, namely the PaRS model, authenticity distinction, and ontological distinction. At present, there are no criteria to clearly favor one of these accounts, although PaRS has problems explaining the results of the similarity task, and the explanation solely based on the authenticity distinction begs the question why authenticity would be more salient in some conditions. More studies are needed to tease apart these alternative explanations. In particular, it would be useful to factor in individual experiences with the tested items to assess the role of representational similarity.

## Ethics statement

This study was carried out in accordance with the recommendations of Ethics Committee at the University of Finance and Management in Warsaw. The Committee reviewed and approved the study design as well as the protocol. All participating children's parents provided written informed consent for their children's participation, in accordance with the Declaration of Helsinki. Children provided oral consent for participation in the study.

## Author contributions

AT contributed to the development of the study hypothesis, the study design, data analysis, drafted the manuscript, discussed the results, and literature, and approved the final version of the manuscript for submission.

### Conflict of interest statement

The author declares that the research was conducted in the absence of any commercial or financial relationships that could be construed as a potential conflict of interest.

## Footnotes

^1^I use the term similarity-based in reference to selections of more similar items, because the underlying process of those selections is not debated.
