# Supplementary material for: Ontological Constraints in Children's Inductive Inferences: Evidence From a Comparison of Inferences Within Animals and Vehicles
Source: Front Psychol. 2018 Apr 30;9:520. doi: 10.3389/fpsyg.2018.00520 (PMC5937141; doi:10.3389/fpsyg.2018.00520)
Supplement: Supplementary file 1 [file Table_1.docx]

*Tarlowski A (2018) Ontological Constraints in Children’s Inductive Inferences: Evidence From a Comparison of Inferences Within Animals and Vehicles. Front. Psychol. 9:520. doi: 10.3389/fpsyg.2018.00520*

**Appendix 1.**

List of items included in the study.

| Type of task | Objects with the feature* | Objects with no feature* |
| --- | --- | --- |
| *Animals* |  |  |
| 12 training (feedback) trials | Dogs | Ice and clouds |
| 12 test (no feedback) trials | Dragonfly  Grasshopper  Ant  Ladybird  butterfly  octopus  squid  Frog  Fish x 2  Snake x 2 | Plush lion  Plush raccoon  Plush bear  Plush sheep  Plush bunny  Plush cat  Plush tiger  Plush rocking horse  Rubber bear  Wooden elephant  Wooden rocking horse  Wooden bear |
| *Artifacts* |  |  |
| 12 training (feedback) trials | Passenger cars | Stones |
| 12 test (no feedback) trials | Ship  Fishing boat x2  Motorcycle x 2  Yacht  Digger  Steam locomotive  Glider  Passenger plane  Helicopter x 2 | Wooden bobtail  Wooden flatbed  Wooden dump truck x 2  Wooden van  Wooden tractor  Metal tow truck  Metal dump truck  Plastic dump truck  Plastic locomotive  Plastic tractor  Lego van |
