# Supplementary material for: Ontological Constraints in Children's Inductive Inferences: Evidence From a Comparison of Inferences Within Animals and Vehicles
Source: Front Psychol. 2018 Apr 30;9:520. doi: 10.3389/fpsyg.2018.00520 (PMC5937141; doi:10.3389/fpsyg.2018.00520)
Supplement: Supplementary file 2 [file Data_Sheet_1.pdf]

Tarlowski A (2018) Ontological Constraints in Children's Inductive Inferences: Evidence From a Comparison of Inferences Within Animals and Vehicles. Front. Psychol. 9:520. doi: 10.3389/fpsyg.2018.00520

Appendix 2.

Instruction for the experimenter.

Experimenter's own words are marked with " "

Descriptions and guidelines for the experimenter are marked with [ ]

The program's recordings are marked with < >

1. *Induction task.*

"We will now talk about what various things have inside. For example, what is inside a wallet?" [try to get the child to name money, cards, coins etc.]

"What is inside a fridge?" [elicit any form of food].

"What do people have inside?" [expect any internal organ, if there is no response point to the chest and ask "What is inside here and beats?"]

"If we want to see what is inside a fridge, what do we have to do?" [elicit "Open"]

"Can we see what is inside a person?" [Elicit "No", if there is a "yes" say "With our own eyes we can?"].

"That is true, we can't, and if the doctor wants to check if the heart is healthy can she do it?"

"Yes she can because she has a special device. Such devices are called detectors. When we want to find out what various things have inside, and we can't see it, then we use a detector. I will show you how the detector works" [show the detector] "This detector detects metal. Look, I have two envelopes here. " [show the envelopes] "There is a piece of metal in one of them." [show the metal to the child]. "We hide the metal in one of them. Now mix the envelopes, as I turn around. I will not look." [the child shuffles the envelopes] "Do I see

where the metal is? No I don't. It is inside. But the detector will help me find it. It will indicate which envelope has the metal inside". [Scan both objects. For one, there will be beeping and light.] "It's here! I could not see, which envelope had the metal, but the detector showed me". [Allow the child to try buy switching roles. Important! Both the experimenter and the child have to scan both objects even if the metal is found in the first one.]

"Now, on a tablet, we will use the detector to check what is inside various things.

[start the program, record child's name, images of particles will appear on the screen.]

"Now I will tell you about particles. Particles are very small bits, they are so small, that one can't see them. Look, these are the drawings of various particles. These particles are inside various things. If the particles can't be seen, how can we know which things have them inside?"

[elicit "Use the detector"]

"Yes! We can use the detector"

"Now choose one picture. We will see which things have such particles inside"

[the child selects one of the 3 images. <These particles are called {blicks}. I wonder which things have {blicks} inside’>

"So these are {blicks}" [repeat the recording, prompt the child to touch on the vertical line that appeared on the screen.]

WARM-UP STARTS

[Two photos of objects appear on the screen. Please note! Never name the objects in the pictures, refer to them as "thing, this", If the child names the objects, ignore that. If she asks, say that you will tell at the end].

"We have two things here. One of them has {blicks} inside, but we don't know which one, because {blicks} can't be seen. Guess what has {blicks} inside. Touch with your finger to check which one has {blicks} inside."

[the child chooses and receives feedback from the program <Yes! This one has {blicks} inside!>, or <No! The other one has {blicks} inside!">]

"See! The detector has shown us what has {blicks} inside. But it will not always show us, sometimes we have to guess ourselves".

[Prompt the child to touch on the vertical line. Two objects appear again]

"And now which one has {blicks} inside?"

[The child makes the selection, objects disappear, there is no feedback from the program]

"Look, this time the detector did not show us, which one has {blicks} inside, but I think you chose the right one/ it was the other one"

[Prompt the child to move to the next trial. Provide feedback on trials where there is no detector]. [At the end of this task circles appear in the screen. Their number corresponds to the number of correct responses.]

<Look how many you have found!> [Repeat praise].

THE END OF WARM-UP.

[2 remaining particles appear on the screen, repeat the selection and introduction of the particle]

FIRST EXPERIMENTAL TASK STARTS.

[The procedure is the same as in warm-up. However there is one key difference relating to feedback. ATTENTION! Give feedback in Warm-up only. In tasks 2 and 3 feedback must not be given on any account! If you do this the task will be invalid! Instead of giving feedback say "This time the detector didn't show us. We will find out at the end"].

[second experimental task starts with the image of the remaining particle, and proceeds in the same way].

2. *Similarity task*

"We will now play the game about things that are similar. Some things look very similar, but there are some that look completely different. We will look for things that are similar. Look! This is a frame. " [show the frame] "We will place things that look similar in this frame."

"Look, I am putting the candle inside the frame. I also have a carrot and a coconut here. Which is more similar to the candle, the carrot or the coconut? Put the one that looks similar to the candle inside the frame."

[expect the carrot]

"Great! The candle and the carrot are similar so we placed them together in the frame. Now look, I am putting an orange in the frame. I also have a ball and a pen. Which is more similar to the orange, the pen or the ball? Put the thing that looks similar to the orange into the frame"

[expect the ball]

"Great! The orange and the ball are similar so we've put them together in the frame".

"And now we will play a bit differently. I have something mysterious inside this envelope. I won't tell you what it is. I will name it, just for fun."

[pretend to think of a name]

"I will call it a tul. Let it be called a tul. There is a mysterious tul in the envelope. I will place the tul under our frame. Now I will show you two things. Guess, which one is more similar to the tul".

[present a round piece of cloth and a square piece of cardboard. Encourage the child to guess and to place the chosen object in the frame. Cloth is the correct answer. If the child selects the wrong object, correct.]

"No this is not more similar to the tul, this is" or "Yes, this is more similar to the tul"

[In both cases the cloth needs to end up in the frame. Repeat with two additional pairs. If the child makes an error in one of those additional pairs (one piece of cloth and one dissimilar object, repeat the initial pair, and loop, until 3 consecutive correct responses. The first can of course be wrong. If the remaining two are correct, end the presentation. The game always ends with showing the child the mystery tul -which ends up being a piece of cloth, similar to the ones in the three pairs.]

"See! This is my mystery tul. Now we are going to do the same on a tablet. In this game we have various mystery objects that have funny names".

[The rest of the task is on a tablet. The procedure is exactly the same as in the induction task, except for the wording of the following instructions/prompts from the program and the experimenter]

<I have something mysterious here. I call it a {blick}. Guess what is similar to the {blick}>

<Yes this is similar to the {blick}> <No, the other one is similar to the {blick}>

"We have two things here. One of them is similar to the {blick}, but we don't know which one. Guess which one is similar to the {blick}. Touch with your finger to check which one is similar to the {blick}."

"See! The frame has shown us what is similar to the {blick}. But it will not always show us, sometimes we have to guess ourselves".

"And now which one is similar to the {blick}?"

“Look, this time the frame did not show us, which is similar to the {blick}, but I think you chose the right one/ it was the other one”
